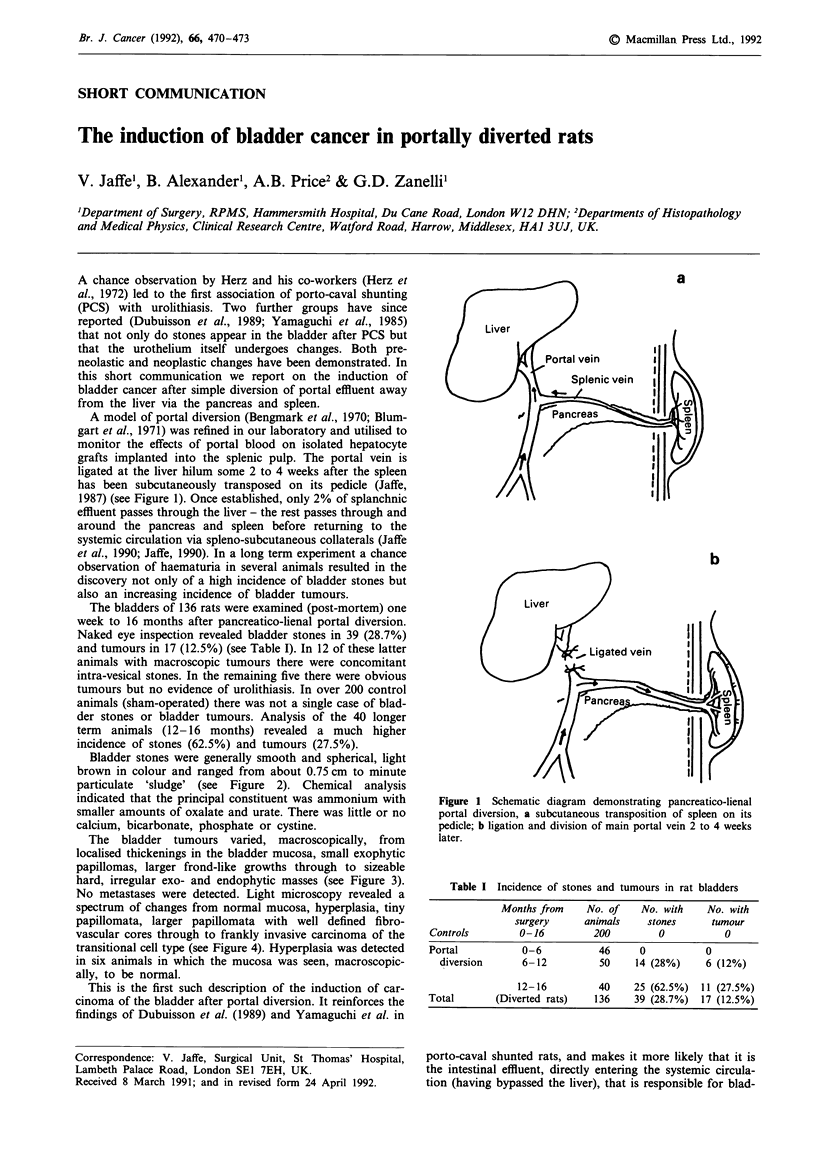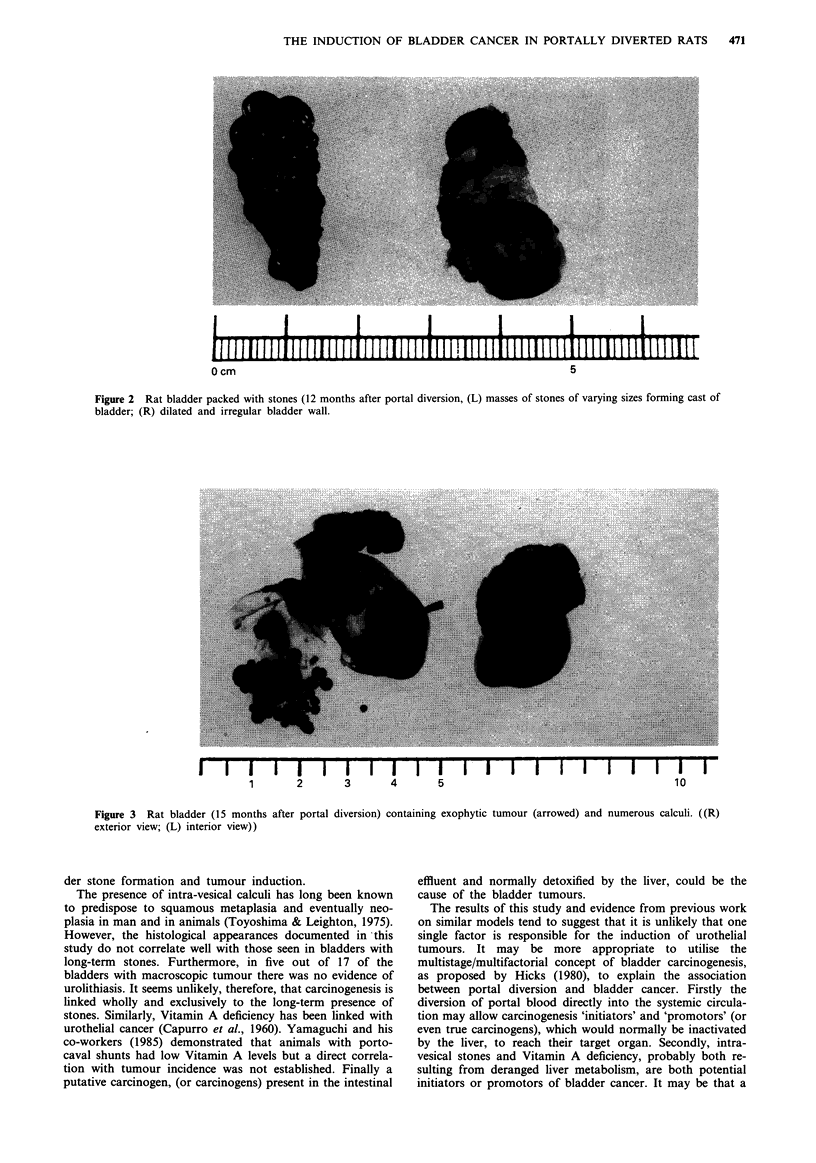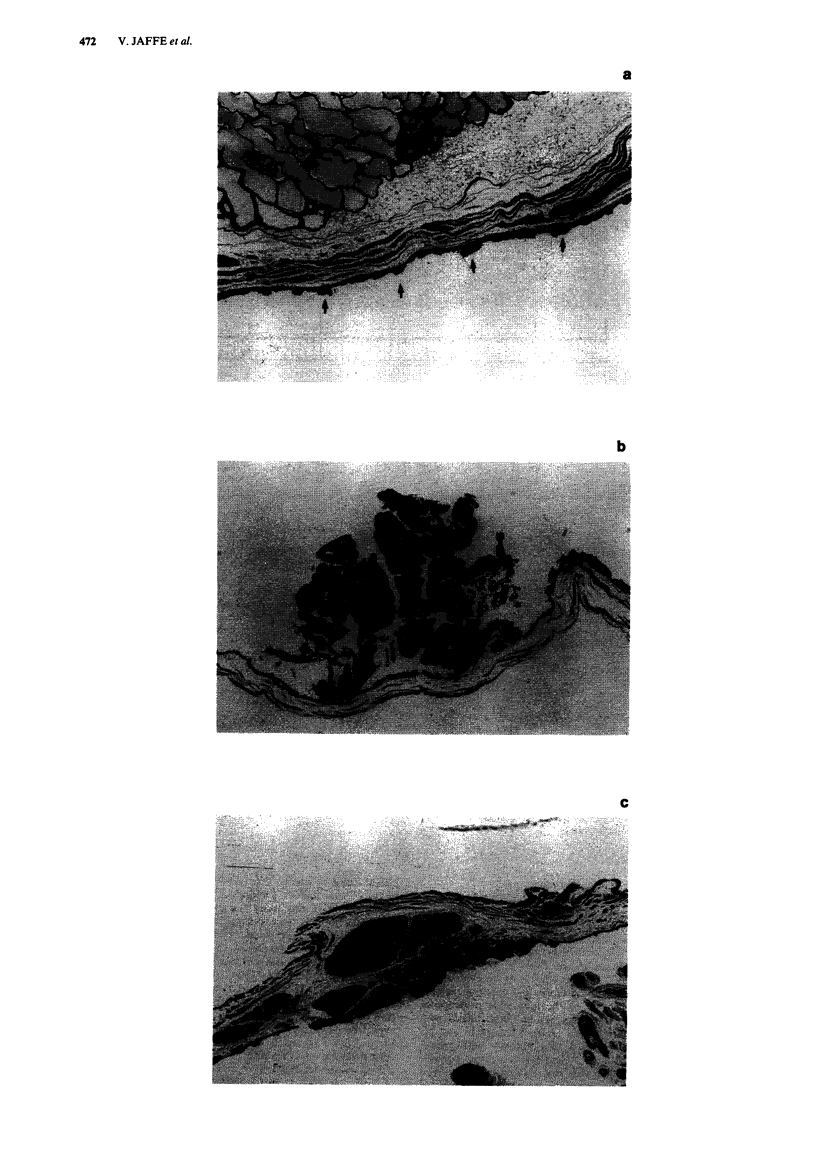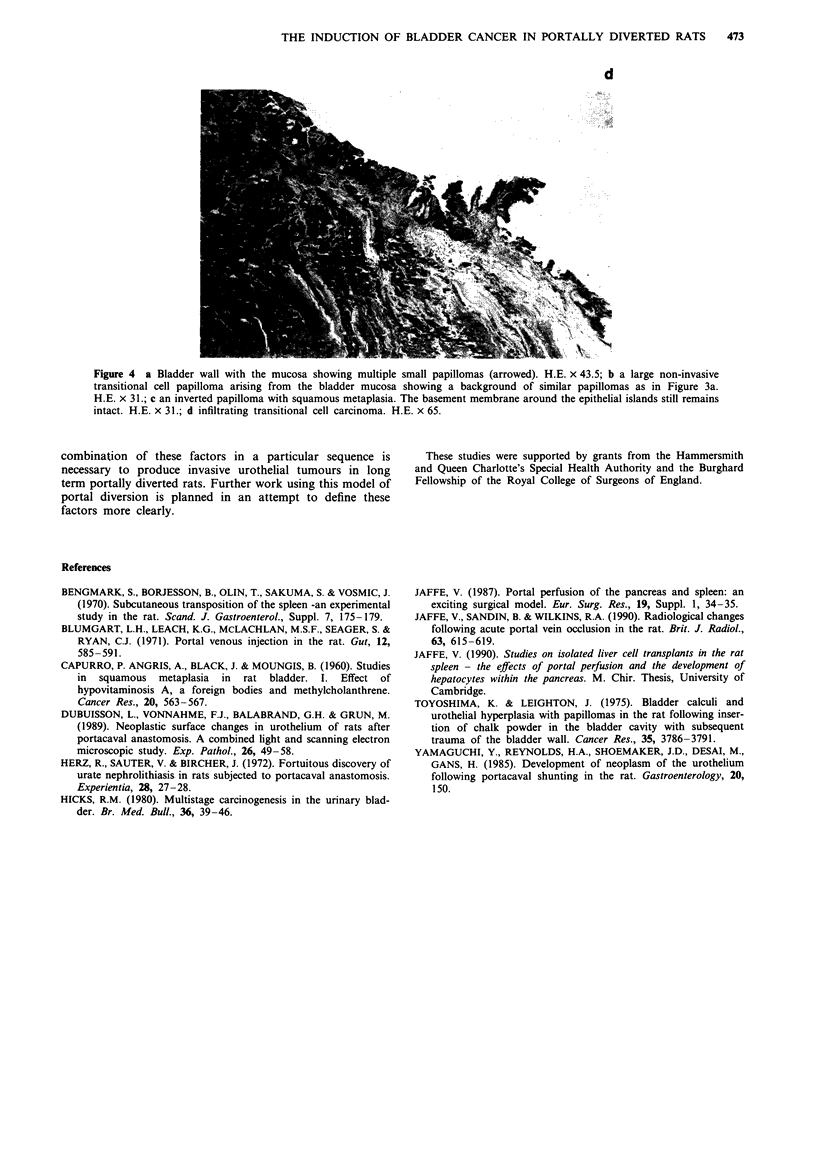# The induction of bladder cancer in portally diverted rats.

**DOI:** 10.1038/bjc.1992.297

**Published:** 1992-09

**Authors:** V. Jaffe, B. Alexander, A. B. Price, G. D. Zanelli

**Affiliations:** Department of Surgery, RPMS, Hammersmith Hospital, London, UK.

## Abstract

**Images:**


*Br. J. Cancer* (1992), **66**, 470–473 © Macmillan Press Ltd., 1992

SHORT COMMUNICATION

### The induction of bladder cancer in portally diverted rats

V. Jaffe[1], B. Alexander[1], A.B. Price[2] & G.D. Zanelli[1]

[1]*Department of Surgery, RPMS, Hammersmith Hospital, Du Cane Road, London W12 DHN;* [2]*Departments of Histopathology and Medical Physics, Clinical Research Centre, Watford Road, Harrow, Middlesex, HA1 3UJ, UK.*

A chance observation by Herz and his co-workers (Herz *et al.*, 1972) led to the first association of porto-caval shunting (PCS) with urolithiasis. Two further groups have since reported (Dubuisson *et al.*, 1989; Yamaguchi *et al.*, 1985) that not only do stones appear in the bladder after PCS but that the urothelium itself undergoes changes. Both pre-neolastic and neoplastic changes have been demonstrated. In this short communication we report on the induction of bladder cancer after simple diversion of portal effluent away from the liver via the pancreas and spleen.

A model of portal diversion (Bengmark *et al.*, 1970; Blumgart *et al.*, 1971) was refined in our laboratory and utilised to monitor the effects of portal blood on isolated hepatocyte grafts implanted into the splenic pulp. The portal vein is ligated at the liver hilum some 2 to 4 weeks after the spleen has been subcutaneously transposed on its pedicle (Jaffe, 1987) (see Figure 1). Once established, only 2% of splanchnic effluent passes through the liver – the rest passes through and around the pancreas and spleen before returning to the systemic circulation via spleno-subcutaneous collaterals (Jaffe *et al.*, 1990; Jaffe, 1990). In a long term experiment a chance observation of haematuria in several animals resulted in the discovery not only of a high incidence of bladder stones but also an increasing incidence of bladder tumours.

The bladders of 136 rats were examined (post-mortem) one week to 16 months after pancreatico-lienal portal diversion. Naked eye inspection revealed bladder stones in 39 (28.7%) and tumours in 17 (12.5%) (see Table I). In 12 of these latter animals with macroscopic tumours there were concomitant intra-vesical stones. In the remaining five there were obvious tumours but no evidence of urolithiasis. In over 200 control animals (sham-operated) there was not a single case of bladder stones or bladder tumours. Analysis of the 40 longer term animals (12–16 months) revealed a much higher incidence of stones (62.5%) and tumours (27.5%).

Bladder stones were generally smooth and spherical, light brown in colour and ranged from about 0.75 cm to minute particulate 'sludge' (see Figure 2). Chemical analysis indicated that the principal constituent was ammonium with smaller amounts of oxalate and urate. There was little or no calcium, bicarbonate, phosphate or cystine.

The bladder tumours varied, macroscopically, from localised thickenings in the bladder mucosa, small exophytic papillomas, larger frond-like growths through to sizeable hard, irregular exo- and endophytic masses (see Figure 3). No metastases were detected. Light microscopy revealed a spectrum of changes from normal mucosa, hyperplasia, tiny papillomata, larger papillomata with well defined fibrovascular cores through to frankly invasive carcinoma of the transitional cell type (see Figure 4). Hyperplasia was detected in six animals in which the mucosa was seen, macroscopically, to be normal.

This is the first such description of the induction of carcinoma of the bladder after portal diversion. It reinforces the findings of Dubuisson *et al.* (1989) and Yamaguchi *et al.* in porto-caval shunted rats, and makes it more likely that it is the intestinal effluent, directly entering the systemic circulation (having bypassed the liver), that is responsible for blad-

**Figure 1** Schematic diagram demonstrating pancreatico-lienal portal diversion, **a** subcutaneous transposition of spleen on its pedicle; **b** ligation and division of main portal vein 2 to 4 weeks later.

**Table I** Incidence of stones and tumours in rat bladders

| | *Months from surgery* | *No. of animals* | *No. with stones* | *No. with tumour* |
|---|---|---|---|---|
| *Controls* | *0–16* | *200* | *0* | *0* |
| Portal | 0–6 | 46 | 0 | 0 |
| diversion | 6–12 | 50 | 14 (28%) | 6 (12%) |
| | 12–16 | 40 | 25 (62.5%) | 11 (27.5%) |
| Total | (Diverted rats) | 136 | 39 (28.7%) | 17 (12.5%) |

Correspondence: V. Jaffe, Surgical Unit, St Thomas' Hospital, Lambeth Palace Road, London SE1 7EH, UK.
Received 8 March 1991; and in revised form 24 April 1992.

**Figure 2** Rat bladder packed with stones (12 months after portal diversion, (L) masses of stones of varying sizes forming cast of bladder; (R) dilated and irregular bladder wall.

**Figure 3** Rat bladder (15 months after portal diversion) containing exophytic tumour (arrowed) and numerous calculi. ((R) exterior view; (L) interior view))

der stone formation and tumour induction.

The presence of intra-vesical calculi has long been known to predispose to squamous metaplasia and eventually neoplasia in man and in animals (Toyoshima & Leighton, 1975). However, the histological appearances documented in this study do not correlate well with those seen in bladders with long-term stones. Furthermore, in five out of 17 of the bladders with macroscopic tumour there was no evidence of urolithiasis. It seems unlikely, therefore, that carcinogenesis is linked wholly and exclusively to the long-term presence of stones. Similarly, Vitamin A deficiency has been linked with urothelial cancer (Capurro *et al.*, 1960). Yamaguchi and his co-workers (1985) demonstrated that animals with portocaval shunts had low Vitamin A levels but a direct correlation with tumour incidence was not established. Finally a putative carcinogen, (or carcinogens) present in the intestinal effluent and normally detoxified by the liver, could be the cause of the bladder tumours.

The results of this study and evidence from previous work on similar models tend to suggest that it is unlikely that one single factor is responsible for the induction of urothelial tumours. It may be more appropriate to utilise the multistage/multifactorial concept of bladder carcinogenesis, as proposed by Hicks (1980), to explain the association between portal diversion and bladder cancer. Firstly the diversion of portal blood directly into the systemic circulation may allow carcinogenesis 'initiators' and 'promotors' (or even true carcinogens), which would normally be inactivated by the liver, to reach their target organ. Secondly, intravesical stones and Vitamin A deficiency, probably both resulting from deranged liver metabolism, are both potential initiators or promotors of bladder cancer. It may be that a a
b
c d

**Figure 4** **a** Bladder wall with the mucosa showing multiple small papillomas (arrowed). H.E. × 43.5; **b** a large non-invasive transitional cell papilloma arising from the bladder mucosa showing a background of similar papillomas as in Figure 3a. H.E. × 31.; **c** an inverted papilloma with squamous metaplasia. The basement membrane around the epithelial islands still remains intact. H.E. × 31.; **d** infiltrating transitional cell carcinoma. H.E. × 65.

combination of these factors in a particular sequence is necessary to produce invasive urothelial tumours in long term portally diverted rats. Further work using this model of portal diversion is planned in an attempt to define these factors more clearly.

These studies were supported by grants from the Hammersmith and Queen Charlotte's Special Health Authority and the Burghard Fellowship of the Royal College of Surgeons of England.

### References

BENGMARK, S., BORJESSON, B., OLIN, T., SAKUMA, S. & VOSMIC, J. (1970). Subcutaneous transposition of the spleen -an experimental study in the rat. *Scand. J. Gastroenterol.*, Suppl. 7, 175–179.

BLUMGART, L.H., LEACH, K.G., MCLACHLAN, M.S.F., SEAGER, S. & RYAN, C.J. (1971). Portal venous injection in the rat. *Gut*, **12**, 585–591.

CAPURRO, P. ANGRIS, A., BLACK, J. & MOUNGIS, B. (1960). Studies in squamous metaplasia in rat bladder. I. Effect of hypovitaminosis A, a foreign bodies and methylcholanthrene. *Cancer Res.*, **20**, 563–567.

DUBUISSON, L., VONNAHME, F.J., BALABRAND, G.H. & GRUN, M. (1989). Neoplastic surface changes in urothelium of rats after portacaval anastomosis. A combined light and scanning electron microscopic study. *Exp. Pathol.*, **26**, 49–58.

HERZ, R., SAUTER, V. & BIRCHER, J. (1972). Fortuitous discovery of urate nephrolithiasis in rats subjected to portacaval anastomosis. *Experientia*, **28**, 27–28.

HICKS, R.M. (1980). Multistage carcinogenesis in the urinary bladder. *Br. Med. Bull.*, **36**, 39–46.

JAFFE, V. (1987). Portal perfusion of the pancreas and spleen: an exciting surgical model. *Eur. Surg. Res.*, **19**, Suppl. 1, 34–35.

JAFFE, V., SANDIN, B. & WILKINS, R.A. (1990). Radiological changes following acute portal vein occlusion in the rat. *Brit. J. Radiol.*, **63**, 615–619.

JAFFE, V. (1990). *Studies on isolated liver cell transplants in the rat spleen – the effects of portal perfusion and the development of hepatocytes within the pancreas.* M. Chir. Thesis, University of Cambridge.

TOYOSHIMA, K. & LEIGHTON, J. (1975). Bladder calculi and urothelial hyperplasia with papillomas in the rat following insertion of chalk powder in the bladder cavity with subsequent trauma of the bladder wall. *Cancer Res.*, **35**, 3786–3791.

YAMAGUCHI, Y., REYNOLDS, H.A., SHOEMAKER, J.D., DESAI, M., GANS, H. (1985). Development of neoplasm of the urothelium following portacaval shunting in the rat. *Gastroenterology*, **20**, 150.